# Resilience and its association with post-traumatic stress disorder, anxiety, and depression symptomatology in the aftermath of trauma: a cross-sectional study from Nepal

**DOI:** 10.1192/j.eurpsy.2022.1732

**Published:** 2022-09-01

**Authors:** S. Dhungana, R. Koirala, S. Ojha, S. Thapa

**Affiliations:** 1 Institute of Medicine, Psychiatry, Kathmandu, Nepal; 2 University of Oslo, Division Of Mental Health And Addiction, Kathmandu, Nepal

**Keywords:** Anxiety, Depression, resilience, Post-traumatic stress disorder

## Abstract

**Introduction:**

Resilience is a multidimensional construct. Despite being quoted as protective against mental disorders, it remains largely unexplored in our context.

**Objectives:**

We attempted to explore the role of resilience in the development of various psychiatric symptoms as depression, anxiety and post-traumatic stress disorder following trauma in clinical population in a psychiatry outpatient of a university hospital.

**Methods:**

We interviewed one hundred patients who sought treatment in psychiatry outpatient in a university hospital in Kathmandu, Nepal. We collected sociodemographic and trauma related information using semi-structured interview format. Other instruments used were the World Health Organization Composite International Diagnostic Interview version 2.1 for trauma categorization, the Post-Traumatic Stress Disorder Checklist-Civilian version to measure the post-traumatic stress disorder symptoms, and the 25-item Hopkins Symptom Checklist-25 to assess the level of depression and anxiety symptoms. We used Nepali adapted resilience scale derived from the original Wagnild and Young Resilience scale to measure resilience. We explored the associations between resilience scores and the scores on depression, anxiety and posttraumatic stress disorder using bivariate and multivariate analysis.

**Results:**

Resilience had negative correlations with depression, anxiety, and post-traumatic stress disorder symptoms after adjusting for other variables such as gender, marital status, employment status, socioeconomic status and trauma types which were observed to have significant association in the bivariate analysis.

**Conclusions:**

There was inverse correlation between resilience scores and depression, anxiety, and post-traumatic stress symptoms. Resilience should be considered in studies involving trauma population.

**Disclosure:**

No significant relationships.

